# The complete plastid genome of *Camellia Octopetala* (Theaceae)

**DOI:** 10.1080/23802359.2019.1664951

**Published:** 2019-09-16

**Authors:** Baojian Ye, Xiongde Tu, Dingkun Liu, Shipin Chen, Hui Chen

**Affiliations:** a College of Forestry, Fujian Agriculture and Forestry University, Fuzhou, China;; b Office of Scientific Research and Development, Fujian Agriculture and Forestry University, Fuzhou, China;; c College of Landscape, Fujian Agriculture and Forestry University, Fuzhou, China

**Keywords:** *Camellia octopetala*, plastid genome, phylogeny

## Abstract

*Camellia octopetala* is a native oil tree species in the south of China and is also a unique natural Chinese woody edible oil species. In the study, the complete plastid genome was assembled and annotated, the genome full-length is 156,615 bp, contains a large single-copy (LSC) region with 86,273 bp, a small single-copy (SSC) region with 18,410 bp, two invert repeats (IR) regions with 25,966 bp. The plastid genome contains 135 genes, 90 protein-coding genes, 37 tRNA genes, and 8 rRNA genes. Phylogenetic analysis shows *C. octopetala* sister to *C. crapnelliana* and embedded in *Camellia*.

*Camellia octopetala* belongs to *Camellia* genus which contains about 120 species all over the world (eFloras 2008). China is a *Camellia* hot spot which contains 97 species (76 endemic) and mainly distributed in Yunan Province, Guangxi Province, Guangdong Province, Sichuan Province of China. *Camellia octopetala* is an evergreen microphanerophyte which is mostly discovered in the understory of the forest with high air humidity, south of China. The species is an economic species which is known for its high oil content of its seeds, similar with the *C. oleifera* (Zhang et al. 2017). Here, we present the study which was assembled and characterized the plastid genome of *C. octopelata* as a resource for evolution and breeding research.

The plant sample of *C. octopetala* was collected from Fujian province, China (Youxi, Sanming: 118°15′32″E, 26°22′07″N) and dried into silica gel immediately. The voucher specimen is deposited at the Herbarium of College of Forestry, Fujian Agriculture and Forestry University (specimen code FJFC04028). DNA extraction from fresh leaf tissue and randomly interrupted using the Covaris ultrasonic breaker for library construction. The 350 bp library was prepared with a NEBNext^®^ Ultra TM DNA Library Prep Kit for Illumina (NEB, Ipswich, MA, USA). Genome was sequenced by PE150 by Illumina Novaseq platform, approximately 10 GB data generated. Raw data quality trimmed by NGSQC with default parameters. Complete plastid genome of *C. octopetala* was assembled by SPAdes 3.9.0 (Bankevich et al. 2012) and MITObim v1.8 (Hahn et al. 2013) with default parameters. Assembled chloroplast genome annotation by DOGMA (Wyman et al., 2004) and CpGAVAS (Liu et al. 2012). The annotation result was drawn with OGDRAW (http://ogdraw.mpimp-golm.mpg.de/) (Lohse et al. 2013).

The complete plastid genome sequence of *C. octopetala* (GenBank accession MN095258) was 156,615 bp in length with a large single-copy (LSC) region of 86,273 bp, a small single-copy (SSC) region of 18,410 bp, and a pair of inverted repeats (IR) regions of 25,966 bp. Complete chloroplast genome contains 135 genes. There were 90 protein-coding genes, 37 tRNA genes, and 8 rRNA genes. The complete genome GC content was 37.3%. To study the phylogenetic relationship of *C. octopetala* with other members of Theaceae, 19 species (five species of *Camellia*, two species of *Polyspora*, one species of *Tutcheria*, two species of *Pyrenaria*, one species of *Apterosperma*, two species of *Schima*, one species of *Franklinia*, two species of *Gordonia*, three species of *Stewartia*) of Theaceae (Yu et al. 2017), all are from NCBI GenBank. The sequences were aligned by MAFFT v7.307 (Katoh and Standley 2013) and phylogenetic tree constructed by RAxML (Stamatakis et al. 2008) (Figure 1). *Camellia octopetala,* sister to *C. crapnelliana*, and genus *Camellia* is monophyletic group embedded in Theaceae with strong bootstrap support.

**Figure 1.**
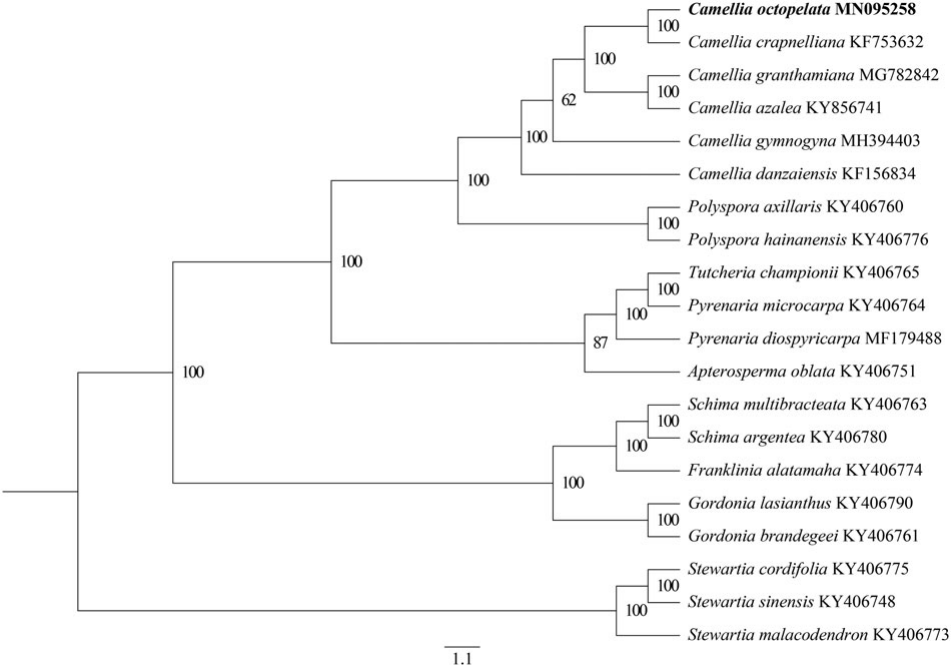
Phylogenetic analysis of 20 species of Theaceae based on plastid genome sequences by RAxML, bootstrap support value near the branch.

## References

[CIT0001] Bankevich A, Nurk S, Antipov D, Gurevich AA, Dvorkin M, Kulikov AS, Lesin VM, Nikolenko SI, Pham S, Prjibelski AD, et al. 2012 SPAdes: a new genome assembly algorithm and its applications to single-cell sequencing. J Comput Biol. 19:455–477. 22506599 10.1089/cmb.2012.0021 PMC3342519

[CIT0002] eFloras. 2008 Camellia octopetala. Flora China. 12:396.

[CIT0003] Hahn C, Bachmann L, Chevreux B 2013 Reconstructing mitochondrial genomes directly from genomic next-generation sequencing reads—a baiting and iterative mapping approach. Nucleic Acids Res. 41:e129. 23661685 10.1093/nar/gkt371 PMC3711436

[CIT0004] Katoh K, Standley DM 2013 MAFFT multiple sequence alignment software version 7: improvements in performance and usability. Mol Biol Evol. 30:772–780. 23329690 10.1093/molbev/mst010 PMC3603318

[CIT0005] Liu C, Shi L, Zhu Y, Chen H, Zhang J, Lin X, Guan X 2012 CpGAVAS, an integrated web server for the annotation, visualization, analysis, and GenBank submission of completely sequenced chloroplast genome sequences. BMC Genomics. 13:715. 23256920 10.1186/1471-2164-13-715 PMC3543216

[CIT0006] Lohse M, Drechsel O, Kahlau S, Bock R 2013 OrganellarGenomeDRAW–a suite of tools for generating physical maps of plastid and mitochondrial genomes and visualizing expression data sets. Nucleic Acids Res. 41:1–7. 23609545 10.1093/nar/gkt289 PMC3692101

[CIT0007] Stamatakis A, Hoover P, Rougemont J 2008 A rapid bootstrap algorithm for the RAxML web servers. Syst Biol. 57:758–771. 18853362 10.1080/10635150802429642

[CIT577776] Wyman S K, Jansen R K, Boore J L. 2004 Automatic annotation of organellar genomes with DOGMA. Bioinformatics. 20:3252–3255. 15180927 10.1093/bioinformatics/bth352

[CIT0008] Yu X-Q, Gao L-M, Soltis DE, Soltis PS, Yang J-B, Fang L, Yang S-X, Li D-Z 2017 Insights into the historical assembly of East Asian subtropical evergreen broadleaved forests revealed by the temporal history of the tea family. New Phytol. 215:1235–1248. 28695680 10.1111/nph.14683

[CIT0009] Zhang W, Zhao Y, Yang G, Tang Y, Xu Z 2017 Characterization of the complete chloroplast genome sequence of *Camellia oleifera* in Hainan, China. Mitochondr DNA B. 2:843–844. 10.1080/23802359.2017.1407687 PMC7799952 33474005

